# Sick-Room Cookery

**Published:** 1908-08-08

**Authors:** Robert Saundby


### Hospital Clinics.

### SICK-ROOM COOKERY.

By PROFESSOR ROBERT SAUNDBY, M.D., LL.D., F.R.C.P.

"Even the young doctors of Berlin learn cooking at the *Lette-Haus*. Special classes for invalid cookery are held on their behalf, and are said to be popular and extremely useful. Certainly doctors whose work is amongst the poor or in country places must often wish they understood something about the preparation of food."—*Home Life in Germany*, by Mrs. Alfred Sidgwick. Pp. 72-3.

The principles of sick-room dietary are sufficiently plain to make it possible to lay them down within the compass of a brief lecture and without going into unnecessary detail. The introduction of milk as the basis of sick-room feeding has been an enormous advance towards simple efficiency, but it should be remembered that milk should always be diluted when given to the sick. Perhaps the best form is that commonly supplied in Germany under the name of Gaertner's fat-milk, which is made by passing milk that has been diluted with 50 per cent. of sterilised water through a separator which divides it into half, thus supplying a milk which contains practically all the fat and half the solids. This milk is then sterilised and sent out in closed bottles for use in the sick-room. It can be obtained in England, but, owing to the process having been patented, the royalty charged has checked its supply. Many people may feel surprised that it should be possible to patent such an obvious process as the foregoing and to put a price on it which has the effect of preventing its use in this country; but this is the consequence of our patent laws as at present administered. In the absence of this convenient preparation we must continue to dilute sterilised milk by adding to it an equal quantity of water, or of some other diluent—barley water is one of the best, as it gives a pleasant flavour to the milk; and although not theoretically so perfect as Gaertner's milk, it is found in practice to work very well. Where the bowels are loose, lime water should take the place of barley water.

If the patient can take a little solid food, milk-roll is preferable to ordinary bread. If toasted it is more easily masticated and it is readily beaten to a pap with milk. It is to be regretted that we have not in this country anything like the same supply that can be obtained in Germany of zwiebacks or rusks suitable for invalids. There these can be obtained sweetened or unsweetened or flavoured in different ways. Most kinds can be purchased in London, but not easily elsewhere, although Huntley and Palmer supply a good German rusk like a tea-cake sliced up and toasted, but relatively expensive. If a teacake is cut up and carefully toasted, but not buttered, it makes a good and cheap substitute.

Eggs beaten up are a simple, highly nutritious, and palatable addition to the diet at this stage. Meat extractives in the form of beef-tea have only a limited value, as they are not food in the proper sense, but merely a convenient and agreeable stimulant gratefully taken by the patient at a time when other foods are distasteful. It is in acute illnesses of short duration, such as pneumonia, that beef-tea is most valuable; a pint of it daily may be prescribed with advantage in such cases. It is only when there is the greatest difficulty in giving any nourishment at all, and only the simplest kinds and smallest quantities of food are tolerated, that we need think of using a solution of albumen. This is made by mixing the white of egg with four times its volume of distilled water and filtering it through tow. If flavoured with a little Liebig it closely resembles in appearance and taste an expensive "fluid meat," which seems to owe much of its popularity to the recommendations of nurses.

Preparations of gelatine are less used than they were a generation ago; they contain a comparatively small amount of nutriment, but they are pleasant to taste, and can be given flavoured either with meat or with wine and sugar. Chicken jelly made by boiling down the carcass of a fowl after the wings and legs have been removed is often liked very much by invalids; good chicken jelly can also be bought ready made at the chemists' shops.

### Sick-room Kitchens.

So far nothing has been suggested that cannot be easily prepared by the nurse or be obtained from the shops, but when the patient's appetite and digestion enable him to go beyond this stage, the skill of the cook is called for, and here there is much need for improvement in the means at our disposal. Only the well-to-do have the facilities for providing sick-room delicacies; in poorer homes not only skill, but time and labour are wanting to prepare the necessary meals. I am therefore of opinion that the provision of sick-room kitchens in our large towns is a distinct want, and that the model for these is to be found where nowadays we go for our examples of so many things, in Germany; in Berlin, Cassel, Aix-la-Chapelle, Bonn, and Vienna there exist institutions which supply meals for the sick at moderate prices. In Berlin the meals are cooked and forwarded within a radius of two kilometres. Since December 1905 the Southwark Invalid Kitchen has been working with the same object, although not quite on the same basis. The objection to the system of the Southwark Sick Kitchen is that it is too much of a charity. Dinners which cost 5d. are supplied for 1d. As I have no wish to add to the number of pauperising institutions already existing, I cannot say that I wish to see more sick kitchens started on these terms, but I should like to see many established on the German model, where meals are supplied at the cost of from 3d. to 2s. 6d. For 3d. can be obtained at the kitchen either half a litre of bouillon with a piece of meat, or one litre of thin oatmeal gruel with an egg, or half a litre of soup with meat and green vegetables or potatoes. The cheaper articles must be fetched by the purchaser, but any of the following are sent home: (1) Roast meat with green vegetables or potato purée, 6d.; (2) the choice of thick or clear soup followed by meat and green vegetables and potato purée, or one litre of soup with chicken and compôte, 1s.; (3) for 1s. 6d. the purchaser gets three portions—chicken or meat soup (clear or thick), roast meat with green vegetables or potato purée, light pudding and compôte; (4) for 2s. 6d. the dinner is much the same, but there is a choice of beef, chicken, or veal (boiled or roast), and both green vegetables and potatoes are included, as well as some light sweet and compôte. Single dishes to be fetched by the purchaser are sold at corresponding prices: A portion of meat and chicken, 1s. 3d.; of vegetables, 6d.; light pudding, 3d.; compôte, 3d. Meat or fruit jelly can also be obtained.

The institution from whose report for 1907 I have taken the above information is called the Society for Sick Cookery in connection with the Evangelical Church Benevolent Society (Verein für Krankenküchen im Anschluss an den Evangelisch-kirchlichen Hilfsverein). During 1907 no fewer than 61,788 portions were supplied, an average of 170 daily, at a total cost of over £2,000. Unfortunately even this institution is not entirely self-supporting; although it received £1,325 10s. 11d. in payments, there was a loss of £773 odd on the year's transactions. I cannot but think that with a little care in administration this gap might be filled up and the income made to balance the expenditure, provided that the scheme were properly supported by members of the medical profession, who could by their patronage ensure a sufficiently large demand for the meals to enable them to be supplied at a minimum cost. I should be sorry to appear to disparage the good work that is being done by the Southwark Sick Kitchen, which during 1907 supplied 11,749 dinners to 565 cases. These dinners were all of the same price—namely, one penny—and consisted of (*a*) chicken, mutton, or rabbit with Yorkshire pudding and vegetables; (*b*) boiled fish with sauce and potatoes; (*c*) beef-tea, mutton, or chicken broth; (*d*) milk pudding or custard pudding; (*e*) jelly. Unquestionably it is a great boon for the doctors and district nurses and visitors among very poor people to be able to order such good meals at so low a price, but it would surely be possible for the people to pay something more like the cost price.

Among minor points of sick-room cookery may be mentioned infusing tea with boiling milk instead of boiling water; serving vegetables as purées, and fruit either mashed up or after having been passed through a hair-sieve, so as to separate indigestible seeds and skins; taking care that any fish is free from bones, and that meat is tender, and that there are no hard or tough pieces which might be bolted. If the patient's teeth are defective all meat should be minced finely before it is cooked, and it is probably always better to begin for the first day or two with minced meat, even when the teeth are numerous and in good order.

### Diet for Diabetics.

Cooking for the sick in chronic illness is a more difficult art, especially where, as in diabetes or gout, a number of articles of food are forbidden. Many, especially diabetics, suffer from the unpalatable monotony of the food supplied to them. It is unfair and unreasonable to expect a diabetic to live upon "beef and greens." The necessary restrictions are, after all, not such as should prevent a varied and appetising diet being supplied for him if those who have the catering to do exercise a little imagination. It is quite true that the department of sweets is closed to him except a certain number of fruits which may be stewed and sweetened with saccharine, notably cranberries and green gooseberries; but there are a great number of vegetables which are allowable in moderate quantities, such as early green peas, French beans, cauliflower, spinach, asparagus, radishes, lettuce, onions, endive, tomatoes, and cucumbers. It is usual now to allow from 4 to 6 oz. of potatoes daily according as they are old or new. A great many dishes can be made for diabetics in the form of salads with fish, oysters, lobster, crab, shrimps, pickled anchovies, eggs, and cheese. It is now recognised as a mistake to give diabetics large quantities of meat, as tending to favour the occurrence of acidosis; dishes made from these lighter articles should take the place of meat, at any rate, at two of the three daily meals. Owing to the necessary absence or strict limitation of bread, there is a difficulty in giving a diabetic a sufficiently nutritious diet, and the deficit has to be made up by the use of fat. Consequently in cooking the food of a diabetic, fats should be used in various ways in the form of fat bacon, butter, cream, and oil, and considerable quantities of these can be taken when they are judiciously mixed with the food in cooking. I append a few dishes suitable for diabetic patients.

Spanish Soup.—Into hot fat put parsley, a little onion, tomatoes (without skins or seeds), and other permitted vegetables not cut up too small, scraps of mutton and poultry, and steam the whole tender. Add the broth and meat from a boiled sheep's head, and serve the whole in a tureen.

Swiss Eggs.—Spread 2 oz. of butter over the bottom of a flat dish, and lay upon it six thin slices of Swiss cheese; break six eggs and put them upon the cheese with the yolks whole; sprinkle over them salt, pepper, finely-chopped parsley, and grated Swiss cheese. Bake the whole in a hot oven for ten or twelve minutes.

Cheese Trifle.—Three oz. of grated cheese, three yolks of eggs, and the whites beaten to snow. Melt some butter, pepper, salt, and a cupful of cream, and mix with cheese and eggs. Pour the whole into a pan and bake for fifteen minutes. Serve hot.

Cheese Omelette.—One egg, 2 oz. of grated Parmesan cheese, a tablespoonful of milk. Beat the egg well, and mix it with the milk. Stir in the cheese and fry it for twenty minutes in a well-buttered pan.

Oyster Omelette.—Take the oysters out of their shells, remove their beards, and chop their bodies up very fine, adding three drops of anchovy sauce. Beat up the yolks and whites of three eggs separately. Mix the yolks with the oysters, add pepper and salt and a little finely-chopped parsley. Melt 1½ oz. of butter in a pan. Mix the white of egg with the oysters and fry the whole some minutes in the butter.

Tomato Soufflé.—Take the insides of six to eight tomatoes and rub them through a hair sieve, add salt, pepper, and lemon juice, and the yolks of two eggs, and mix well. Beat the white of three eggs until it is stiff, and stir it carefully. Put the whole in a buttered pan and fry it quickly over a good fire. When ready, sprinkle some grated Parmesan cheese over it.

Fish Salad.—Cut fresh broiled fish, freed from bones and skin, into square pieces half-an-inch thick, mix them with cold mustard sauce with capers, and put them on a flat dish. Over them lay anchovies, capers, pickled mushrooms, gherkins, hard-boiled egg cut in slices, and lettuce.

Meat Salad.—Cold boiled or broiled beef or veal cut into squares and mixed with the sauce described below, capers, chopped anchovies, and gherkins. Put them on a dish and garnish with anchovies, hard boiled egg and lettuce.

The Sauce.—Some hard-boiled yolks of eggs to be rubbed up with vinegar, oil, mustard, parsley, and shredded onion.

### What to Avoid.

It may be well to say a few words upon the kind of food which should be avoided in sickness. In acute disease simplicity should be the rule. There can be no reasonable objection, so long as the patient is seriously ill, to a uniformity of diet of which a healthy person might complain. It is only when convalescence commences that the appetite of the patient may tempt him to run risks, though more often it is the unwise solicitude of his friends, who press upon him articles of food he is better without. It shourd be remembered that acute illness abolishes, or at least diminishes, the activity of the glands which secrete the juices by which our food is digested, and that during convalescence these functions are only regained slowly; so that food should be given in a form that is easily assimilated, and the organs of digestion should not be taxed to the normal extent until some weeks have elapsed.

As one of the effects undoubtedly promoted by cooking is to effect, at least partially, those changes by which the food is made assimilable, it is a good rule to give only cooked food; but to this rule there are some exceptions, of which milk is the most important. The juice of fresh fruit is another. Oysters are more readily digested when raw than when cooked, but, owing to the possible danger of typhoid infection, which must be admitted to be greater when the digestive powers are feeble, it is better not to give them uncooked to patients who are convalescing from acute diseases.

This rule would, of course, exclude all uncooked fruit and vegetables, but it need not prevent the use of fruit juice either as a cooling drink or sucked from the fruit as in the case of grapes. A sample of a preparation of grape juice was recently brought to my notice as a beverage for invalids, but it had a disagreeably sweet and slightly metallic flavour, which suggested that saccharin had been added to it in order to check fermentation.

We must regard as unsuitable for patients recovering from acute diseases, pork, veal, goose, duck, any smoked or salted meat or fish, salmon, mackerel, eels, lobster, and crab. Probably few people in this country would propose to give any of these articles to a convalescent patient, but veal enters into the list of dishes supplied by the Berlin Society, whose work has been already described, and I have known German doctors recommend "scraped ham" or "goose" as the first solid food to a patient recovering from acute illness! It is also hardly necessary to point out that condiments, pickles, sauces, and pepper are undesirable. Light China tea should be preferred to the coarser kinds from India and Ceylon, and is preferably infused with boiling milk. Coffee should be largely diluted with milk. Stimulants are, as a rule, unnecessary, and, as they retard a feeble digestion, should be excluded as harmful; only in exceptional cases are they indicated.

